# Caveolin-1 Attenuates Excitotoxic Signaling by Regulating NMDA, AMPA, and Kainate Receptor-Mediated Calcium Influx in Hippocampal Neuronal Cultures

**DOI:** 10.3390/ijms27125637

**Published:** 2026-06-22

**Authors:** Swapna Kannothum Kandy, Madhura Milind Nimonkar, Suravi Sasmita Dash, Prashanth N. Vashista, Bhupesh Mehta, Yogananda S. Markandeya

**Affiliations:** Department of Biophysics, National Institute of Mental Health and Neuroscience (NIMHANS), Bengaluru 560029, India; swapna.bajesh@gmail.com (S.K.K.); memadhura5@gmail.com (M.M.N.); sasmitadash66@gmail.com (S.S.D.); prashanthnvashista@gmail.com (P.N.V.)

**Keywords:** caveolin-1, glutamate, excitotoxicity, hippocampal neurons, NMDA receptor, AMPA receptor, kainate receptor, calcium signaling, reactive oxygen species, mitochondrial membrane potential

## Abstract

Glutamate excitotoxicity is a critical pathological mechanism underlying neuronal death in ischemic stroke, epilepsy, and neurodegenerative diseases. Caveolin-1 (Cav-1), a structural protein of caveolae membrane microdomains, has emerged as a potential modulator of neuronal survival, yet its precise mechanisms in excitotoxicity remain incompletely understood. In this study, we investigated the role of Cav-1 in regulating glutamate-induced calcium dysregulation, reactive oxygen species (ROS) generation, and mitochondrial dysfunction in primary hippocampal neurons. Using Cav-1 overexpression (Cav-1OE) and Cav-1 knockdown (Cav-1KD) approaches, we demonstrate that Cav-1OE significantly attenuates glutamate-stimulated intracellular Ca^2+^ elevation, reduces ROS generation, and prevents mitochondrial membrane potential (Ψ_m_) depolarization. Further investigation revealed that Cav-1OE reduces, while Cav-1KD enhances, calcium responses mediated by NMDA, AMPA, and KA receptors. These findings establish that Cav-1 functionally attenuates excitotoxic signaling by negatively regulating ionotropic glutamate receptor-mediated Ca^2+^ influx.

## 1. Introduction

Glutamate excitotoxicity represents a central pathological mechanism in acute neurological injuries, including ischemic stroke, traumatic brain injury, and epilepsy, as well as chronic neurodegenerative disorders such as Alzheimer’s disease, Parkinson’s disease, and amyotrophic lateral sclerosis [1,2]. Excessive activation of ionotropic glutamate receptors—particularly N-methyl-D-aspartate receptors (NMDARs), α-amino-3-hydroxy-5-methyl-4-isoxazolepropionic acid receptors (AMPARs), and kainate receptors (KARs) triggers pathological Ca^2+^ influx into neurons, initiating a cascade of deleterious events including mitochondrial dysfunction, ROS generation, and ultimately cell death [1].

The hippocampus, a brain region critical for learning and memory, is particularly vulnerable to excitotoxic injury due to its high density of glutamate receptors and metabolic demands [3]. Hippocampal CA1 pyramidal neurons are especially susceptible to ischemic damage and excitotoxic insults, making them an important model system for investigating neuroprotective mechanisms [4]. Understanding the molecular determinants that regulate glutamate receptor function and downstream excitotoxic signaling is therefore essential for developing therapeutic strategies against neurological disorders.

Cav-1, the principle structural protein of caveolae-specialized plasma membrane microdomains enriched in cholesterol and sphingolipids, has emerged as a key regulator of cellular signaling and membrane organization [5,6,7]. In the central nervous system, Cav-1 is expressed in neurons, astrocytes, and endothelial cells, where it modulates diverse processes including synaptic transmission, and ion channel function [8,9,10]. The caveolin scaffolding domain (CSD) of Cav-1 interacts with numerous signaling molecules, including G-protein coupled receptors, receptor tyrosine kinases, and ion channels, thereby regulating their localization, trafficking, and activity [11].

Accumulating evidence suggests that Cav-1 plays an important role in various models of neurological injury, Cav-1 expression is upregulated following cerebral ischemia, and genetic deletion of Cav-1 exacerbates ischemic brain damage and blood–brain barrier disruption [12,13]. Furthermore, Cav-1 has been implicated in modulating oxidative stress responses and mitochondrial function in neurons [14]. However, the precise molecular mechanisms by which Cav-1 exerts its putative neuroprotective effects against excitotoxicity remain incompletely understood.

Previous studies have suggested that Cav-1 may influence calcium homeostasis and mitochondrial function during excitotoxic stress, but conflicting reports exist regarding whether Cav-1 acts primarily at the plasma membrane level by regulating glutamate receptor function or directly modulates mitochondrial calcium handling. Additionally, the relative contributions of different ionotropic glutamate receptor subtypes to Cav-1-mediated [Ca^2+^]_i_ have not been systematically investigated.

In this study, we employed primary hippocampal neuron cultures with genetic modulation of Cav-1 expression (Cav-1OE and Cav-1KD) to dissect the mechanisms underlying Cav-1-mediated attenuation of excitotoxic signaling against glutamate excitotoxicity. Using live-cell fluorescence imaging, we systematically examined the effects of Cav-1 on glutamate-induced [Ca^2+^]_i_ elevation, ROS generation, and Ψ_m_ depolarization. Critically, we investigated the role of Cav-1 in mitochondrial calcium uptake or acts upstream by modulating plasma membrane glutamate receptor function. Our findings reveal that Cav-1 reduces ROS formation primarily through negative regulation of NMDA-, AMPA-, and KA receptor-mediated Ca^2+^ influx at the plasma membrane, thereby preventing downstream oxidative stress and mitochondrial dysfunction. These results provide mechanistic insight into Cav-1-mediated attenuation of excitotoxic signaling and identify Cav-1 as a potential therapeutic target for excitotoxic neuronal injury.

## 2. Results

### 2.1. Caveolin-1 OE and KD Effectively Modulate Protein Levels in Hippocampal Neurons

**Figure 1:**
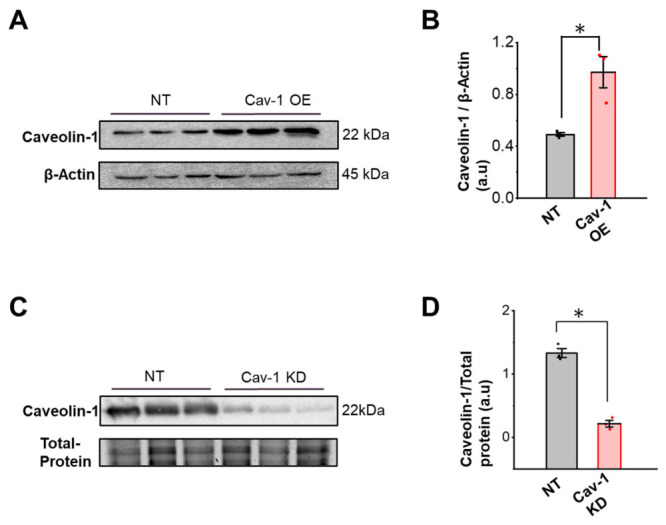
Effects of Cav-1OE and Cav-1KD on hippocampal neurons. (**A**) Western blot image for the expression of Cav-1 in NT and Cav-1OE neurons with β-actin as the loading control. (**B**) Dot plot representing quantification of Cav-1 expression normalized to β-actin. (**C**) Western blot image for the expression of Cav-1 in NT and Cav-1KD neurons with total protein as the loading control. (**D**) Dot plot representing quantification of Cav-1 expression normalized to total protein. Data are represented as mean ± S.E.M. from 3 experiments, ∗ *p* < 0.05.

To investigate the role of Cav-1 in glutamate excitotoxicity, we first established hippocampal neuron cultures with altered Cav-1 expression levels. Primary hippocampal neurons were transfected with either *Cav1*-mCherry fusion construct (Cav-1OE) (Supplementary Figure S1) or transduced with lentiviral shRNA targeting *Cav1* (Cav-1KD) (Supplementary Figure S3). Western blot analysis at DIV 13 confirmed successful modulation of Cav-1 protein expression (Figure 1A,C; Supplementary Figures S4 and S5). Densitometric quantification revealed that Cav-1OE neurons exhibited approximately ~97% increase in Cav-1 protein levels compared to non-transfected (NT) controls (Figure 1B), while Cav-1KD neurons showed approximately ~84% reduction in Cav-1 expression relative to scrambled shRNA (Sc shRNA) controls (Figure 1D). These expression changes were statistically significant and consistent across multiple independent culture preparations, validating the efficacy of our genetic modulation approaches.

### 2.2. Cav-1OE Attenuates Glutamate-Induced [Ca^2+^]_i_ Rise

**Figure 2:**
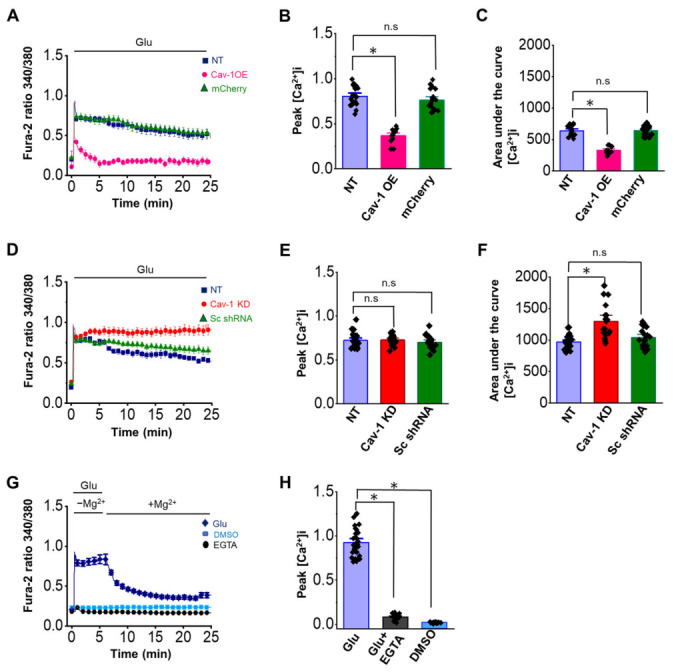
Cav-1OE reduces excitotoxic [Ca^2+^]_i_ rise in hippocampal neurons. (**A**–**C**) Representative Fura-2 AM fluorescence traces showing [Ca^2+^]_i_ dynamics in neurons exposed to glutamate (chronic exposure, 25 min) in Mg^2+^-free conditions for NT, Cav-1OE, and mCherry-only transfected neurons. (**D**) Average Ca^2+^ response curves upon glutamate exposure (1.8 mM extracellular Ca^2+^, no Mg^2+^) in NT (_▀_, *n* = 20), Cav-1OE (●, *n* = 15), and Sc shRNA transduced (▲, *n* = 20) neurons. (**E**,**F**) Dot plots quantifying peak [Ca^2+^]_i_ levels and AUC for each group as indicated. (**G**,**H**) Average Ca^2+^ response curves and dot plot showing peak Ca^2+^ response in NT neurons under 5 min glutamate stimulation (no Mg^2+^), followed by 20 min perfusion with Mg^2+^-containing buffer. Conditions include: Glu (♦, *n* = 31), EGTA-treated (1 mM; ●, *n* = 16), and DMSO vehicle (_▀_, *n* = 20). Data are presented as mean ± SEM from ≥3 independent experiments. * *p* < 0.05; n.s. = not significant.

Excessive Ca^2+^ influx through ionotropic glutamate receptors is the primary trigger of excitotoxic neuronal injury. To determine whether Cav-1 modulates glutamate-induced Ca^2+^ responses, we performed live-cell ratiometric Ca^2+^ imaging using Fura-2 AM in hippocampal neurons with altered Cav-1 expression. Application of 100 µM glutamate plus 10 µM glycine induced robust [Ca^2+^]_i_ elevation in all neuronal populations (Figure 2). However, the magnitude of Ca^2+^ response was significantly modulated by Cav-1 expression levels. Peak Ca^2+^ amplitude and area under the curve (AUC)—in other words total calcium—were analyzed independently to distinguish maximal acute intracellular Ca^2+^ elevation following receptor activation from sustained intracellular Ca^2+^ load over time. Peak amplitude reflects the maximal receptor-induced Ca^2+^ response, whereas AUC incorporates both response magnitude and duration. Quantitative analysis revealed that Cav-1OE neurons exhibited a 55% reduction in peak Ca^2+^ response (0.36 ± 0.01; *n* = 15), compared to NT controls (0.8 ± 0.02; *n* = 20) and a 52% reduction in mock-transfected controls (0.76 ± 0.02; *n* = 20) (Figure 2B; Supplementary Figure S2). Conversely, Cav-1KD neurons displayed no significant difference in glutamate-induced peak Ca^2+^ amplitude relative to Sc shRNA (control 0.8256 ± 0.06, *n* = 18, Cav-1KD 0.8375 ± 0.06, *n* = 19) (Figure 2D,E). The AUC analysis of Ca^2+^ responses over the 20 min recording period showed similar trends, with Cav-1OE significantly reducing total [Ca^2+^]_i_ load by 48% (330.4 ± 25.0) relative to both NT (641 ± 18.4) and mCherry controls (644.8 ± 14.9) (Figure 2C). The AUC in Cav-1KD (AUC: 1296.4 ± 63.7) was significantly increased by 34% compared to NT (AUC 967 ± 23.3) and 25% in Sc shRNA transduced neurons (1037.06 ± 37.77), (Figure 2D,F). These findings demonstrate that Cav-1 negatively regulates glutamate-induced [Ca^2+^]_i_ elevation in hippocampal neurons.

Glutamate activates both ionotropic (iGluR) and metabotropic (mGluR) glutamate receptors [15]. To determine whether this suppression involved mGluRs, experiments were repeated in Ca^2+^-free buffer supplemented with 1 mM EGTA. Neurons were stimulated with 100 μM glutamate in the presence of 1.8 mM Ca^2+^ and 0 mM Ca^2+^ (with 1 mM EGTA). Our results show that glutamate stimulation significantly increased both peak Ca^2+^ levels (0.93 ± 0.02, *n* = 31) and total [Ca^2+^]_i_ (AUC 657.26 ± 18.30) in the presence of 1.8 mM Ca^2+^. In contrast, no changes in the Ca^2+^ response were observed when extracellular Ca^2+^ was absent (EGTA peak Ca^2+^ 0.08 ± 0.01, AUC 257.70 ± 4.75, *n* = 16; Figure 2G,H), suggesting no involvement of mGluRs. Additionally, in the absence of glutamate (vehicle: peak Ca^2+^ 0.02 ± 0.001, AUC 257.70 ± 29.41, *n* = 20), the Ca^2+^ response was significantly lower compared to neurons treated with glutamate, highlighting the role of iGluRs (Supplementary Figure S8). Together, these findings demonstrate that Cav-1OE potently suppresses both the amplitude and duration of excitotoxic [Ca^2+^]_i_ elevation by modulating Ca^2+^ influx through iGluRs and Cav-1KD enhances Ca^2+^ response to glutamate during excitotoxicity.

### 2.3. Caveolin-1 Regulates ROS Generation During Excitotoxicity

**Figure 3:**
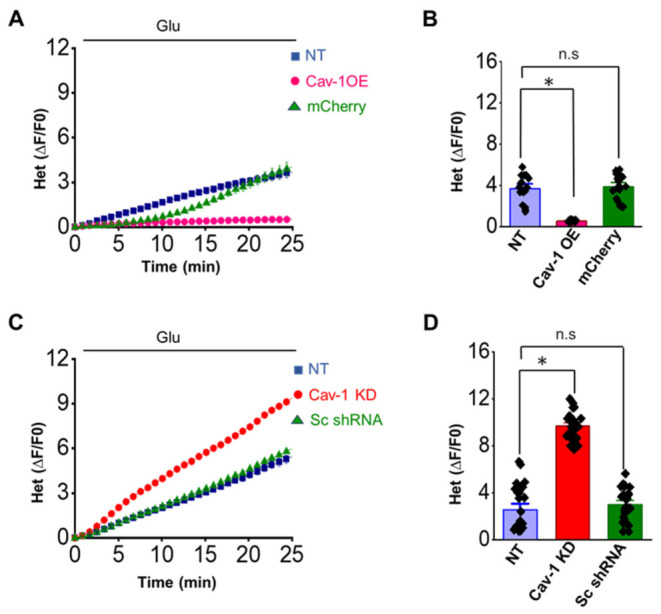
Cav-1OE attenuates, whereas Cav-1KD aggravates, ROS generation during glutamate-induced excitotoxicity in hippocampal neurons. *(***A**) Average ROS traces of Het fluorescence intensity indicating ROS formation in response to glutamate stimulation in NT (_▀_, *n* = 17), Cav-1OE (●, *n* = 15), and mCherry-only transfected (▲, *n* = 19) neurons. (**B**) Dot plot quantifying mean ROS levels across groups. (**C**) Average ROS traces indicating ROS formation in response to glutamate stimulation in NT (_▀_, *n* = 28), Cav-1KD (●, *n* = 35), and Sc shRNA transduced (▲, *n* = 33) neurons. (**D**) Dot plot quantifying mean ROS levels across groups. Data are presented as mean ± SEM from ≥3 independent experiments. * *p* < 0.05; n.s. = not significant.

Excessive Ca^2+^ influx during excitotoxicity triggers ROS generation through multiple mechanisms, including activation of NADPH oxidases and mitochondrial dysfunction [16]. To assess whether Cav-1-mediated attenuation of Ca^2+^ responses translates to reduced oxidative stress, we measured ROS production using the fluorogenic probe dihydroethidium (DHE) during glutamate stimulation. Glutamate application (100 µM + 10 µM glycine) induced progressive ROS accumulation in all neuronal groups, as evidenced by increasing DHE fluorescence over time. However, Cav-1 expression levels profoundly influenced the magnitude of ROS generation. Cav-1OE neurons exhibited significantly attenuated ROS production, with peak DHE fluorescence reduced. Cav-1 OE neurons exhibited significantly lower ROS levels (0.53 ± 0.07, *n* = 15) (Figure 3A,B), indicating a ~7-fold reduction in oxidative stress compared to NT (3.68 ± 0.31; *n* = 17). In contrast, ROS was robustly increased by 3.7-fold (9.54 ± 0.21, *n* = 35) in Cav-1KD than in NT (2.55 ± 0.35, *n* = 28) (Figure 3C,D). Time-course analysis revealed that the protective effect of Cav-1OE was evident throughout the glutamate exposure period, with significantly lower ROS levels maintained from 5 min onward (Figure 3B). Vehicle control traces are provided in Supplementary Figure S6. Conversely, Cav-1KD neurons displayed accelerated kinetics of ROS accumulation. These results establish that Cav-1 has negative regulation on ROS formation during excitotoxcity in hippocampal neurons.

### 2.4. Caveolin-1 Modulates Mitochondrial Membrane Potential Depolarization

**Figure 4:**
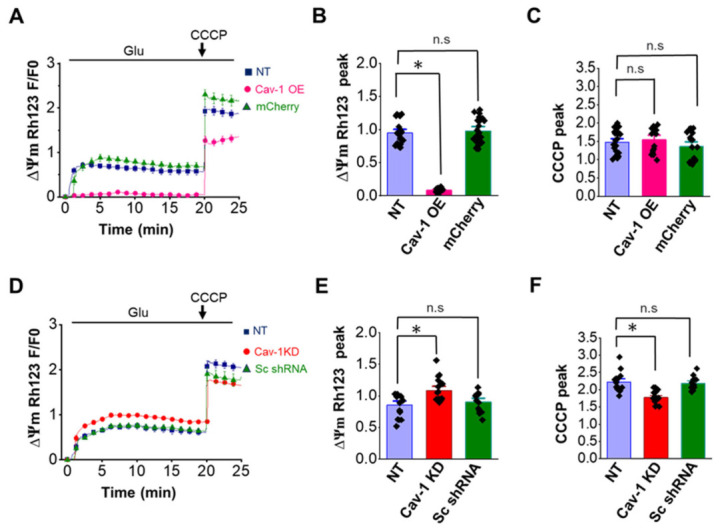
Cav-1OE attenuates, while Cav-1KD potentiates, glutamate-induced Ψ_m_ depolarization in hippocampal neurons. (**A**) Average Rh123 fluorescence traces showing changes in Ψ_m_ during chronic glutamate exposure (100 µM) in NT (_▀_, *n* = 22), Cav-1OE (●, *n* = 16), and mCherry-only (▲, *n* = 21) transfected neurons. Arrowheads indicate the time of glutamate application. (**B**,**C**) Dot plots quantifying mean Ψ_m_ and the mean response to CCCP (used as a positive control for mitochondrial depolarization) in each group. (**D**) Average Rh123 fluorescence traces in NT (_▀_, *n* = 17), Cav-1KD (●, *n* = 18), and Sc shRNA (▲, *n* = 14) transduced neurons. (**E**,**F**) Dot plots quantifying mean ΔΨ_m_ and the mean response to CCCP. Arrowheads indicate the time of glutamate application. Data are presented as mean ± SEM from ≥3 independent experiments. * *p* < 0.05; n.s. = not significant.

Mitochondrial dysfunction, characterized by loss of Ψ_m_, is a critical downstream consequence of excitotoxic Ca^2+^ overload and ROS generation [5,9,10,17]. To determine whether Cav-1-mediated reduction in Ca^2+^ influx and ROS production preserves mitochondrial function, we monitored Ψ_m_ using the potentiometric dye rhodamine 123 (Rh123) during glutamate stimulation. In NT control neurons, glutamate application (100 µM + 10 µM glycine) induced progressive mitochondrial depolarization, reflected by increasing Rh123 fluorescence due to dye release from depolarized mitochondria (Figure 4A). Cav-1OE neurons exhibited significantly attenuated mitochondrial depolarization, with significant reduction in the peak Rh123 fluorescence. In control (NT) neurons, glutamate triggered a significant increase in Rh123 signal (0.92 ± 0.05, *n* = 22), indicating mitochondrial depolarization (Figure 4A). In contrast, Cav-1OE neurons displayed a ~91.3% reduction in Rh123 fluorescence (0.08 ± 0.01, *n* = 16; Figure 4A,B), with no biphasic or delayed responses, suggesting preserved mitochondrial polarization under excitotoxic stress. Conversely, Cav-1KD neurons displayed exacerbated mitochondrial depolarization, with peak Rh123 fluorescence increase elevated by 27% (1.08 ± 0.04, *n* = 18) relative to NT controls (0.85 ± 0.03, *n* = 17) (Figure 4D,E).

Time-course analysis demonstrated that Cav-1OE delayed the onset and reduced the rate of mitochondrial depolarization throughout the glutamate exposure period (Figure 4B). At the end of each experiment, application of the mitochondrial uncoupler CCCP (1 µM) induced maximal depolarization in all groups, confirming that the observed differences reflected genuine changes in Ψ_m_ rather than variations in dye loading or mitochondrial mass. Twenty minutes after glutamate application, CCCP treatment did not significantly alter Rh123 fluorescence (1.54 ± 0.08, *n* = 16), compared to NT controls (1.47 ± 0.06, *n* = 22) (Figure 4C), suggesting no further mitochondrial depolarization in Cav-1OE neurons. In Cav-1KD neurons, CCCP application showed a significant decrease (20.2%) in Rh123 fluorescence (1.77 ± 0.04, *n* = 18) when compared to the NT controls (2.22 ± 0.03, *n* = 17) (Figure 4F). Supplementary Figure S7 contains vehicle control traces. These findings indicate that Cav-1 preserves Ψ_m_ during glutamate excitotoxicity.

### 2.5. Limited Influence of Caveolin-1 on Mitochondrial Calcium Uptake

**Figure 5:**
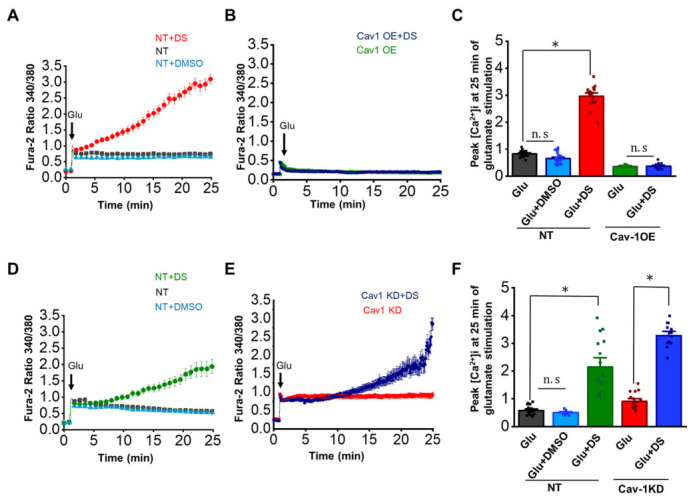
Caveolin-1 expression shows no effect on mitochondrial calcium uptake during glutamate stimulation in hippocampal neurons. (**A**) Average [Ca^2+^]_i_ responses in NT neurons following glutamate application in the presence (red) or absence of the MCU inhibitor DS. Arrowheads indicate the time of glutamate application. (**B**) Average Ca^2+^ traces in Cav-1OE neurons in the presence and absence of DS. (**C**) Dot plot showing the mean [Ca^2+^]_i_ rise post-glutamate stimulation across DS-treated and untreated groups: NT (*n* = 20 treated, *n* = 19 untreated) and Cav-1OE (*n* = 17 treated, *n* = 11 untreated). (**D**) Average Ca^2+^ traces of neurons upon glutamate application in the presence of MCU complex blocker DS and the absence of DS. (**E**) Average Ca^2+^ traces in Cav-1KD neurons in the presence of DS and the absence of DS. Arrowheads indicate the point of application. (**F**) Dot plot representing the mean rise in [Ca^2+^]_i_ after glutamate addition in DS-treated (NT, *n* = 17, Cav-1KD, *n* = 15) and DS non-treated groups (NT, *n* = 17, Cav-1KD, *n* = 18). Data are presented as mean ± SEM from ≥3 independent experiments. * *p* < 0.05; n.s. = not significant.

The preceding results demonstrated that Cav-1 attenuates glutamate-induced cytosolic Ca^2+^ elevation, ROS generation, and mitochondrial depolarization. However, it remained unclear whether Cav-1 acts on mitochondria to modulate mitochondrial Ca^2+^ uptake or whether its effects are entirely mediated through reduced plasma membrane Ca^2+^ influx. To investigate the effect of Cav-1 on mitochondrial calcium sequestration we used the specific MCU inhibitor DS16570511 (DS) [18]. Neurons were incubated with 30 µM DS for 30 min before exposure to 100 µM glutamate. In the NT group, inhibition of the MCU led to a dramatic increase in cytosolic Ca^2+^: the peak [Ca^2+^]_i_ reached 2.96 ± 0.08 (*n* = 20) compared to 0.82 ± 0.02 (*n* = 19) without DS, indicating that mitochondria normally buffer glutamate-induced Ca^2+^ via the MCU (Figure 5A,C). Consistent with previous reports on MCU function in excitotoxic buffering [19], blocking MCU impaired this defense mechanism. In contrast, Cav-1OE neurons showed similarly low Ca^2+^ elevations, whether treated with DS (0.37 ± 0.02, *n* = 17) or untreated (0.36 ± 0.02, *n* = 11) (Figure 5B,C). These data indicate that Cav-1OE lowers cytosolic Ca^2+^ influx to below the threshold required to activate MCU-dependent mitochondrial uptake. Taken together, these findings are consistent with Cav-1OE preserving Ψ_m_ not by altering MCU activity, but by reducing excessive Ca^2+^ entry at the plasma membrane, thereby limiting downstream mitochondrial stress. Similarly, Cav-1KD neurons exhibit a significant increase in cytosolic Ca^2+^ accumulation upon MCU inhibition. DS treatment resulted in a peak Ca^2+^ response of 3.27 ± 0.39 (*n* = 15), whereas neurons without DS showed a lower response (0.91 ± 0.06, *n* = 18) (Figure 5E,F). Likewise, in the NT neurons, exposure to glutamate caused a significant rise in the peak [Ca^2+^]_i_ in the presence of DS (2.12 ± 0.31, *n* =17), when compared to the experiment without DS (0.79 ± 0.02, *n* =17) (Figure 5D,F). These findings indicate that, in Cav-1KD neurons, glutamate-induced Ca^2+^ elevations exceed the buffering capacity of mitochondria when MCU activity is blocked. The rise in [Ca^2+^]_i_ observed in both DS-treated NT and Cav-1KD neurons suggests that Cav-1KD may not modulate MCU function, but instead influences the cytosolic Ca^2+^ entry mechanism that regulates cytosolic Ca^2+^ levels before mitochondrial uptake.

### 2.6. Caveolin-1 Modulates NMDA Receptor-Associated Calcium Responses

**Figure 6:**
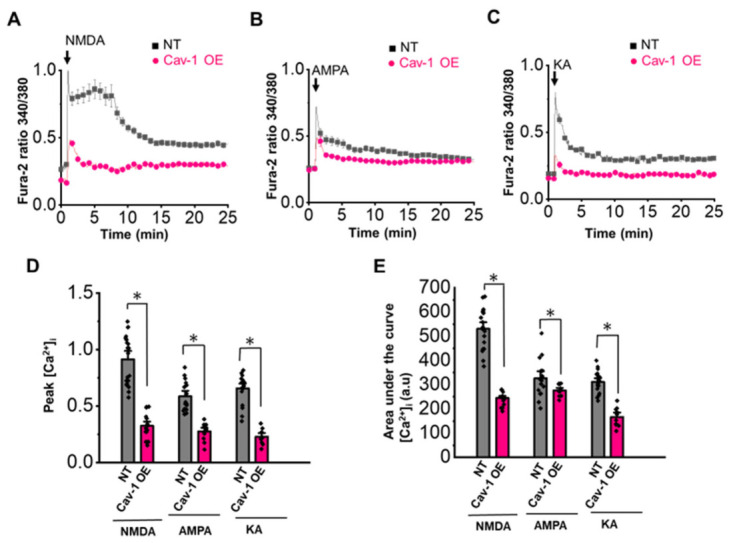
Cav-1OE attenuates NMDA, AMPA, and KA receptor-mediated calcium responses in hippocampal neurons. (**A**–**C**) Average [Ca^2+^]_i_ traces in NT (black) and Cav-1OE (pink) neurons upon stimulation with specific glutamate receptor agonists: NMDA, AMPA, and KA. Arrowheads indicate the time of agonist application. (**D**,**E**) Dot plots representing peak [Ca^2+^ ]_i_ and AUC for each agonist condition in NT and Cav-1OE neurons. Sample sizes: NMDA (NT, *n* = 20; Cav-1OE, *n* = 19), AMPA (NT, *n* = 16; Cav-1OE, *n* = 12), KA (NT, *n* = 19; Cav-1OE, *n* = 10). Data are presented as mean ± SEM from ≥3 independent experiments. * *p* < 0.05.

Given that mitochondrial Ca^2+^ handling is largely unaffected by Cav-1 expression, we believe that Cav-1 may be regulating ionotropic glutamate receptor function at the plasma membrane. To test this hypothesis, we first examined NMDA receptor-mediated Ca^2+^ responses using selective pharmacological stimulation. Application of 50 µM NMDA plus 5 µM glycine in Mg^2+^-free extracellular solution induced robust Ca^2+^ influx in all neuronal populations (Figure 6A). However, Cav-1 expression significantly modulated NMDAR-mediated Ca^2+^ responses. Cav-1OE neurons exhibited a 65% reduction in peak NMDA-induced Ca^2+^ elevation (0.32 ± 0.06) compared to NT controls (0.91 ± 0.04) (Figure 6D). Similarly, the total Ca^2+^ load was significantly decreased in Cav-1OE neurons (AUC 244.86 ± 5.8) relative to NT neurons (530.52 ± 18.2), representing a reduction of 54% (Figure 6E).

Analysis of Ca^2+^ response kinetics revealed that Cav-1OE reduced both the initial rate of Ca^2+^ rise and the sustained plateau phase of NMDAR activation (Figure 6A). These results establish that Cav-1 modulates NMDAR-associated Ca^2+^ influx, thereby attenuating excitotoxic Ca^2+^ signaling.

### 2.7. Caveolin-1 Modulates AMPA and KA Receptor-Associated Calcium Responses

While NMDA receptors are the primary mediators of excitotoxic Ca^2+^ influx, AMPA and KA receptors also contribute to glutamate-induced neuronal injury, particularly through Ca^2+^-permeable receptor subtypes [20,21,22]. To determine whether Cav-1 regulation extends to non-NMDA ionotropic glutamate receptors, we examined Ca^2+^ responses mediated by AMPA and KA receptors. Application of 50 µM AMPA and 50 µM kainate (KA) induced rapid peak Ca^2+^ elevations in hippocampal neurons. Cav-1OE neurons exhibited significantly reduced peak amplitude from 0.59 ± 0.03 to 0.27 ± 0.02 for AMPAR corresponding to a decrease of 54% and from 0.66 ± 0.02 to 0.23 ± 0.02 for KA with a decrease of 65% (Figure 6B–D). Consistent with these findings, the total Ca^2+^ load was also significantly reduced in Cav-1OE neurons relative to NT neurons following both AMPA and KA stimulation. Specifically, Cav-1OE neurons showed a reduction in AUC from 326.33 ± 19.5 to 276.08 ± 6.9 for AMPA, corresponding to a decrease of 15%, and from 311.23 ± 10.1 to 166.52 ± 12.4 for KA, corresponding to a decrease of 46% (Figure 6E).

**Figure 7:**
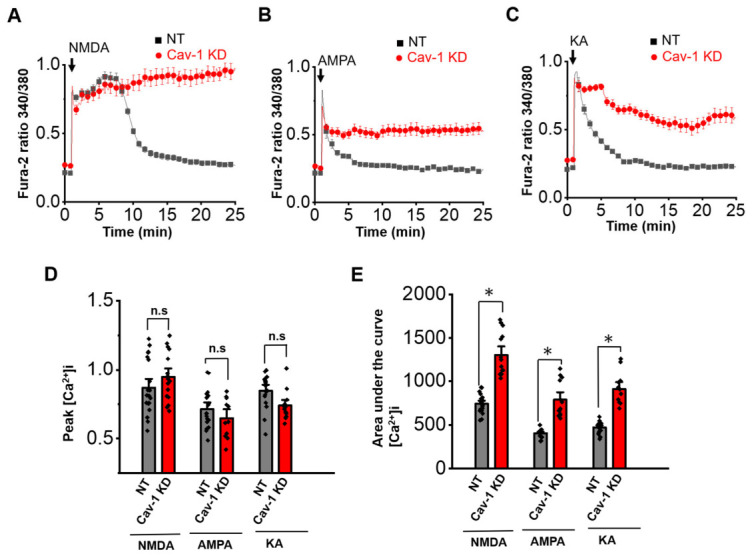
Cav-1KD enhances NMDA, AMPA, and KA receptor-mediated cytosolic Ca^2+^ responses. (**A**–**C**) The average [Ca^2+^]_i_ response in NT (black) and Cav-1KD (red) neurons was stimulated with NMDA (50 μM + 5 μM glycine), 50 μM each of AMPA, and KA for 25 min. The point of GluRs agonist applications is represented as arrowheads. (**D**,**E**) The dot plot of the peak [Ca^2+^]_i_ response and the AUC of NMDA, AMPA, and KA stimulation in NT and Cav-1KD (NMDA: NT, *n* = 21, Cav-1KD, *n* = 16; AMPA: NT, *n* =18, Cav-1KD, *n* = 11; KA: NT, *n* = 18, Cav-1KD, *n* = 14). Data represented as mean ± SEM from 3–4 experiments from 3 batches of neuronal cultures, * *p* < 0.05, n.s. = non-significant.

Cav-1KD neurons did not exhibit a significant increase in peak Ca^2+^ amplitude relative to NT neurons following stimulation with any of the three ionotropic glutamate receptor agonists—NMDA, AMPA, or kainate (peak NMDA: 0.97± 0.04; NT: 0.92 ± 0.04, AMPA; 0.65 ± 0.04; NT: 0.71 ± 0.03 and KA: 0.74 ± 0.02, *n* = 16; NT: 0.84 ± 0.02) (Figure 7A–D). However, the total Ca^2+^ load or AUC was significantly elevated in Cav-1KD neurons compared to NT controls following stimulation with all three agonists (Figure 7E). Specifically, Cav-1KD neurons showed an increase in AUC from 741.99 ± 23.03 to 1305 ± 66.38 for NMDA, corresponding to an increase of 76%; from 404.51 ± 10.61; to 788.19 ± 56.73 for AMPA, corresponding to an increase of 95%; and from 471.50 ± 15.50 to 909.23 ± 53.61 for kainate, corresponding to an increase of 93% (Figure 7E). Overall, these findings indicate that Cav-1 modulates AMPA- and KA receptor-mediated calcium influx during excitotoxicity.

## 3. Discussion

In this study, we provide comprehensive mechanistic evidence that Cav-1 functionally limits glutamate-induced excitotoxic responses in cultured rat hippocampal neurons by negatively regulating ionotropic glutamate receptor function at the plasma membrane. The data from four independent experimental readouts—Ca^2+^ imaging, ROS measurement, mitochondrial membrane potential, and MCU pharmacology—enables a mechanistically coherent model to be constructed and tested against the published literature. Our key findings are: (1) Cav-1OE attenuates, while Cav-1KD enhances, glutamate-induced [Ca^2+^]_i_ elevation, ROS generation, and mitochondrial depolarization; (2) Mitochondrial Ca^2+^ uptake were largely unaffected; and (3) Cav-1 negatively regulates intracellular Ca^2+^ response mediated by NMDA, AMPA and KA receptors in Cav-1OE/KD neurons, respectively. Taken together these results establish that Cav-1-mediated putative neuroprotective effects operate primarily by modulating plasma membrane glutamate receptor function, thereby preventing the initiation of excitotoxic signaling cascades.

In neurons, glutamate activates both ionotropic and metabotropic receptors; however, our calcium chelation experiments indicate that metabotropic glutamate receptors are not involved. Consistent with our earlier reports [22], excitotoxicity-induced intracellular Ca^2+^ responses are biphasic. The primary rapid peak reflects Ca^2+^ influx through ionotropic receptors—NMDA, AMPA, and kainate—while the secondary elevation arises from the release of Ca^2+^ accumulated during the initial peak, either from intracellular organelles or through plasma-membrane channels. Our results indicate that Cav-1 expression regulates the magnitude as well as the kinetics of [Ca^2+^]_i_ response in excitotoxic glutamate stimulation via iGluRs. More importantly, our data demonstrate that Cav-1 predominantly modulates the peak Ca^2+^ response. Specifically, Cav-1OE reduced NMDAR-mediated influx by 54% (AUC), AMPAR-mediated influx by 15%, and KAR-mediated influx by 46%. Conversely, Cav-1KD enhanced NMDAR responses by 76%, AMPAR responses by 95%, and KAR responses by 93% compared to scrambled shRNA controls. The comparable magnitude and bidirectional regulation observed across all three receptor families argues against a subunit-specific mechanism, and instead implicates Cav-1 in a common membrane-organizing role. This coordinated regulation of NMDA, AMPA, and kainate receptor-mediated Ca^2+^ influx by Cav-1 represents the central mechanistic finding of this study.

In the context of the present study, the Cav-1-mediated reduction in the initial ionotropic receptor-gated Ca^2+^ peak is mechanistically significant: by attenuating the primary Ca^2+^ influx through NMDA, AMPA, and kainate receptors, Cav-1OE is predicted to limit the amplitude of the trigger signal that recruits these secondary amplification mechanisms. This upstream attenuation would consequently reduce ER Ca^2+^ store mobilization; lower the Ca^2+^ burden on mitochondria thereby preventing mPTP opening and Ψ_m_ collapse; and diminish downstream ROS generation, consistent with the attenuation of excitotoxic signaling observed across multiple readouts in Cav-1OE neurons across all four experimental paradigms in this study. Importantly, others have suggested that Cav-1 may interact with and modulate various ion channels and receptors through its scaffolding domain [5]; however, direct evidence for Cav-1 regulation of glutamate receptors in neurons has been limited.

The mechanism by which Cav-1 regulates glutamate receptor function likely involves multiple processes. First, Cav-1 may directly interact with glutamate receptor subunits through the CSD, which contains a conserved amino acid sequence (residues 82–101) that binds to caveolin-binding motifs in target proteins [23]. Others have shown that Cav-1 directly interacts with the NR2B subunit of the NMDA receptor in the anterior cingulate cortex, enhancing receptor activity in chronic pain models [24]. In contrast, our results highlight the inhibitory effects of Cav-1 in hippocampal neurons, emphasizing the context-dependent nature of Cav-1 regulation across brain regions and disease states. Such interactions could alter receptor trafficking, surface expression, or channel-gating properties. Second, Cav-1 may indirectly modulate glutamate receptor function by organizing membrane microdomains that influence receptor localization and clustering. Caveolae and lipid rafts serve as platforms for concentrating signaling molecules, and disruption of these microdomains can profoundly affect receptor function [25,26].

The regulation of all three ionotropic receptor classes suggests that Cav-1 may act through a common mechanism affecting multiple glutamate receptor families. Notably, glutamate itself upregulates *Cav1* mRNA and protein expression in hippocampal neurons via KA- and AMPA-receptor activation [27], suggesting a potential feedback loop in which excitotoxic stimulation induces Cav-1 as an endogenous compensatory response. Future studies employing systematic Co-IP, proximity ligation assays, and super-resolution microscopy will be necessary to definitively establish whether Cav-1 directly interacts with glutamate receptor subunits and to map the precise molecular interfaces involved.

The null result from the MCU inhibitor DS16570511 (DS) paradigm is an important outcome of our study. In mCherry control neurons, MCU inhibition elevated peak [Ca^2+^]_i_ from 0.74 to 2.05, confirming robust mitochondrial Ca^2+^ buffering under basal conditions. In Cav-1OE neurons, however, DS treatment produced no significant change in [Ca^2+^]_i_ (0.36 vs. 0.37), indicating that Cav-1OE reduces Ca^2+^ influx to a level below the threshold required to engage MCU-dependent uptake. In Cav-1KD neurons, DS treatment increased peak [Ca^2+^]_i_ from 0.91 to 3.27, confirming that the elevated Ca^2+^ load in Cav-1KD neurons engages, but does not saturate, MCU-dependent buffering. Critically, the DS-induced increment in [Ca^2+^]_i_ was statistically indistinguishable between NT and Cav-1KD neurons, indicating that Cav-1KD does not alter the intrinsic Ca^2+^ uptake capacity of the MCU. It is therefore possible that Cav-1 influences mitochondrial Ca^2+^ homeostasis through MCU independent pathways, or that such effects emerge under different Ca^2+^ loading conditions or in intact tissue. Furthermore, a DMSO vehicle control at the equivalent solvent concentration confirmed no significant difference in Ca^2+^ response kinetics or amplitude compared to untreated neurons (Figure 5A,D), ruling out non-specific solvent effects on the DS result, thus not excluding the possibility that Cav-1 may affect other aspects of mitochondrial physiology.

This finding contrasts with some previous reports suggesting that Cav-1 may directly influence mitochondrial function [28,29]. However, those studies primarily examined Cav-1 effects on mitochondrial ROS production and bioenergetics rather than Ca^2+^ uptake per se. The preservation of Ψ_m_ in Cav-1OE neurons can therefore be attributed to reduced Ca^2+^ and ROS burden on mitochondria, rather than enhanced mitochondrial resilience. This interpretation is consistent with the well-established excitotoxicity model, in which excessive cytosolic Ca^2+^ and ROS overwhelm mitochondrial buffering capacity, leading to mitochondrial Ca^2+^ overload, opening of the mitochondrial permeability transition pore, and collapse of Ψ_m_ [8,15,30]. Cav-1’s upstream regulation of Ca^2+^ entry may therefore suppress Ca^2+^ mediated signaling pathways in excitotoxic conditions.

The reduction in glutamate-induced ROS generation by Cav-1OE (~7-fold reduction vs. NT) and its enhancement by Cav-1KD (3.7-fold increase vs. NT) is the most interesting observation of this study, suggesting that the Cav-1 expression level modulates excitotoxic signaling. The mechanism of ROS attenuation by Cav-1OE is likely multifactorial. First, the 55% reduction in peak [Ca^2+^]_i_ in Cav-1OE neurons would directly decrease Ca^2+^-dependent activation of NADPH oxidases [31], which are major sources of superoxide production during NMDA receptor activation [16]. Second, reduced cytosolic Ca^2+^ elevation is insufficient for mitochondrial Ca^2+^ uptake, thereby preventing mitochondrial ROS production that would otherwise generate from increased electron leak from the respiratory chain and inhibition of antioxidant enzymes [32]. This is consistent with the ~91.3% reduction in Rh123 fluorescence in Cav-1OE neurons, indicating near-complete preservation of Ψ_m_.

The identification of Cav-1 as a constitutive, tonic negative regulator of all three major ionotropic glutamate receptor classes—demonstrated by the bidirectional modulation of Ca^2+^ responses in both Cav-1OE and Cav-1KD neurons—has important implications for excitotoxic neurological disorders [33,34]. Consistent with this hypothesis, previous studies have shown that Cav-1 expression is upregulated following cerebral ischemia, potentially as an endogenous neuroprotective response [35,36]. Some reports have shown that Cav-1KD exacerbates ischemic brain injury and blood–brain barrier disruption, while Cav-1OE or treatment with CSD peptides reduces infarct size and improves functional outcomes [11,37]. Our mechanistic findings provide a molecular explanation for these observations by demonstrating that Cav-1 limits excitotoxic Ca^2+^ influx at the initiating step of the injury cascade.

Interestingly, the role of Cav-1 in neurological disease may be context-dependent. While our results and the existing literature support a probable neuroprotective role for Cav-1 in excitotoxic injury, some studies have reported that Cav-1KD reduces brain injury in models of intracerebral hemorrhage [38]. These apparently contradictory findings may reflect distinct roles of Cav-1 in neurons versus other cell types (e.g., endothelial cells, microglia) or different injury mechanisms (excitotoxicity versus inflammation and blood–brain barrier disruption).

Beyond its role in attenuating excitotoxic signaling in acute injury models, Cav-1 regulation of glutamate receptors may have important implications for synaptic plasticity and learning. NMDA, AMPA, and KA receptors are critical mediators of synaptic transmission and plasticity in the hippocampus [39,40,41]. The observation that Cav-1 negatively regulates these receptors suggests that Cav-1 expression levels could influence synaptic strength and plasticity. Consistent with this possibility, previous studies have shown that Cav-1 modulates synaptic transmission and plasticity in hippocampal neurons [42,43,44]. Cav-1 knockout mice exhibit altered hippocampal synaptic plasticity and behavioral abnormalities [45,46]. Our findings suggest that these effects may be mediated, at least in part, through altered glutamate receptor function. Future studies examining the role of Cav-1 in long-term potentiation and long-term depression will be important for understanding how Cav-1 balances neuroprotection against excitotoxicity with the maintenance of normal synaptic function.

The identification of Cav-1 as a negative regulator of ionotropic glutamate receptors opens new avenues for therapeutic intervention in excitotoxic neurological disorders. Several strategies could be envisioned: (1) gene therapy approaches to enhance Cav-1 expression in vulnerable neuronal populations; (2) small molecule compounds that stabilize Cav-1 or enhance its interaction with glutamate receptors [47]; (3) cell-permeable peptides derived from the CSD that mimic Cav-1 function; and (4) interventions that modulate membrane lipid composition to enhance caveolae formation and function. Some of these approaches have already shown promise in preclinical studies. Treatment with CSD peptides reduces ischemic brain injury and improves outcomes in stroke models [11,48]. Modulation of membrane cholesterol and lipid raft composition has been shown to affect glutamate receptor function and excitotoxicity [49]. Our mechanistic findings provide a strong rationale for further development and testing of these therapeutic strategies.

Important questions remain to be addressed in future studies. First, the precise molecular mechanisms by which Cav-1 regulates glutamate receptor function need to be elucidated, including whether direct protein–protein interactions occur and which receptor subunits are involved. Second, the relative contributions of neuronal versus glial Cav-1 to neuroprotection need to be determined using cell-type-specific genetic approaches. Third, the effects of Cav-1 modulation on neuronal survival and functional outcomes in in vivo models of excitotoxic injury should be systematically evaluated. Finally, the potential for Cav-1-based therapies to be combined with existing pharmacological strategies to attenuate excitotoxic injury (e.g., hypothermia, antioxidants, and glutamate receptor antagonists) should be explored.

This study has several important limitations that should be acknowledged when interpreting the findings. First, all experiments were performed in primary hippocampal neuron cultures, and the findings have not yet been validated in in vivo models of ischemia or neurodegeneration. Such cultures, while informative, do not fully recapitulate the complex cellular interactions and microenvironment of the intact brain. Validation in vivo, particularly in animal models of excitotoxic injury, will be essential to establish the translational relevance of these results. Second, although we demonstrated that Cav-1 regulates Ca^2+^ influx through NMDA, AMPA, and KA receptors, the precise mechanism—whether direct or indirect—remains unresolved and requires further investigation. Third, the use of mixed neuronal and astrocyte cultures introduced additional complexity, and hippocampal neurons were transfected with Cav-1 constructs carrying an mCherry reporter, which precluded the use of propidium iodide-based viability assays due to spectral overlap. Moreover, viral transduction in Cav-1KD experiments and subsequent antibiotic selection markedly reduced the number of available primary hippocampal neurons for analysis, limiting the robustness of conventional viability quantification in heterogeneous cultures. Furthermore, our study does not provide direct evidence for the impact of Cav-1 on neuronal survival, nor does it establish definitive direct interactions between Cav-1 and specific glutamate receptor subtypes; these remain important questions for future investigation. Finally, neuronal protection was inferred indirectly through ROS assays, which, while informative, provide only a surrogate measure of cell viability in excitotoxicity experiments.

## 4. Materials and Methods

### 4.1. Materials

Cell culture chemicals were from Invitrogen, Thermo Fisher Scientific (Waltham, MA, USA). Fluorescent probes Fura-2 AM, Rh123 and Dihydro ethidium (Het) were from Invitrogen, Thermo Fisher Scientific (Waltham, MA, USA). DS16570511 was from Cayman Chemical (Ann Arbor, MI, USA). AMPA was from Tocris, Bio-Techne India Private Limited (Bristol, UK), and NMDA and KA and all other chemicals are from Sigma-Aldrich, Merck, (St Louis, MO, USA).

### 4.2. Animals

All experiments were conducted in compliance with the guidelines set by the Institutional Animal Care and Use Committee (IAEC Ref No: AEC/70/454/BP) at NIMHANS. Post-natal day-0 Wistar rat pups were sourced from the Central Animal Research Facility (CARF) at NIMHANS, Bengaluru, India.

### 4.3. Primary Hippocampal Neuron Culture

Primary hippocampal neurons were isolated from postnatal day-0 (P0) Wistar rat pups as previously described with minor modifications [50]. Briefly, pups were decapitated, and whole brains were rapidly dissected and placed in ice-cold Hanks’ balanced salt solution (HBSS) containing (in mM): 135 NaCl, 5.3 KCl, 4.0 NaHCO_3_, 5.0 KH_2_PO_4_, 3.0 Na_2_HPO_4_, 1.2 CaCl_2_, 1.2 MgCl_2_, and 10 glucose (pH 7.4). Hippocampi were carefully micro-dissected and transferred to Ca^2+^- and Mg^2+^-free HBSS supplemented with 0.25% (*w*/*v*) trypsin. Enzymatic digestion was performed for 20 min at 37 °C in a humidified incubator.

Following digestion, hippocampal tissue was rinsed twice with Ca^2+^- and Mg^2+^-containing HBSS to inactivate trypsin, then washed once with Eagle’s Minimum Essential Medium (EMEM) supplemented with 10% (*v*/*v*) heat-inactivated horse serum (HS), 5% (*v*/*v*) fetal bovine serum (FBS), 100 µg/mL streptomycin, 100 U/mL penicillin, and 1% (*v*/*v*) B-27 supplement (Thermo Fisher Scientific, Waltham, MA, USA). Tissue was mechanically dissociated by gentle trituration using fire-polished Pasteur pipettes. Dissociated cells were plated at a density of 1.5–2.0 × 10^5^ cells onto 16 mm poly-L-lysine-coated glass coverslips (BD Biosciences, San Jose, CA, USA) containing EMEM supplemented with 7.5% HS, 5% FBS, and antibiotics. Cultures were maintained at 37 °C in a humidified atmosphere containing 5% CO_2_ and 95% air.

After 24 h, the plating medium was replaced with maintenance medium consisting of EMEM supplemented with 5% HS and 5% FBS. On day 3 in vitro (DIV 3), cultures were treated with 10 µM cytosine β-D-arabinofuranoside (Ara-C; Sigma-Aldrich, St. Louis, MO, USA) for 24 h to inhibit glial proliferation. On DIV 4, Ara-C-containing medium was removed and replaced with fresh maintenance medium. Subsequently, half-media changes were performed every 3 days until experiments were conducted at DIV 12–14. 

### 4.4. Caveolin-1 Overexpression and Knockdown

Cav-1OE: At DIV 8, hippocampal neurons were transfected with either the *Cav1*-mCherry fusion construct or an empty mCherry vector (mock control) using Lipofectamine 3000 transfection reagent (Thermo Fisher Scientific, Waltham, MA, USA) according to the manufacturer’s protocol. Briefly, 1 µg of plasmid DNA and 2 µL of Lipofectamine 3000 were complexed in Opti-MEM (Thermo Fisher Scientific, Waltham, MA, USA) and applied dropwise to neuronal cultures. Transfection efficiency (~15–20% of neurons) was confirmed by visualizing mCherry fluorescence under an epifluorescence microscope 72 h post-transfection. All experiments utilizing Cav-1OE neurons were performed 72 h post-transfection (DIV 11).

Cav-1KD: shRNA-mediated KD of *Cav-1* was achieved by transducing DIV 8 hippocampal neurons with lentiviral particles encoding *Cav1*-specific short hairpin RNA (shRNA) (Santa Cruz Biotechnology, Dallas, TX, USA (Cat. No.: sc-29241-V)) at a multiplicity of infection (MOI) of 5. Lentiviral particles carrying non-targeting Sc shRNA (Santa Cruz Biotechnology, Dallas, TX, USA; Cat. No.: sc-108080) were used as negative controls. Following 48 h of transduction, stable KD was established by antibiotic selection with 3 µg/mL puromycin (Sigma-Aldrich, St. Louis, MO, USA) for 3 consecutive days. Puromycin-resistant cells were maintained in puromycin-free maintenance medium for an additional 48 h before experimentation.

Effective modulation of Cav-1 protein expression was confirmed by Western blot analysis (Section 2.3). Only cultures demonstrating > 70% Cav-1OE or > 60% Cav-1KD relative to NT or Sc shRNA controls, respectively, were used for subsequent [Ca^2+^]_i_, Ψ_m_, and ROS measurements.

### 4.5. Western Blot Analysis

Caveolin-1 protein expression was quantified by Western blot analysis [51]. Hippocampal neurons (NT controls, Cav-1OE, Cav-1KD, and Sc shRNA controls) were lysed in ice-cold RIPA buffer (50 mM Tris-HCl pH 7.4, 150 mM NaCl, 1% Triton X-100, 0.5% sodium deoxycholate, 0.1% SDS) supplemented with protease inhibitor (Sigma-Aldrich, St. Louis, MO, USA). Protein concentrations were determined using the Pierce BCA Protein Assay Kit (Thermo Fisher Scientific, Waltham, MA, USA).

Equal amounts of protein (20–30 µg per lane) were resolved on 12% SDS-PAGE gels or TGX Stain-Free Fast Cast gels (Bio-Rad Laboratories, Hercules, CA, USA) and electrophoretically transferred onto 0.45 µm PVDF membranes (Millipore, Burlington, MA, USA) using a semi-dry transfer system. Membranes were blocked for 1 h at room temperature in Tris-buffered saline containing 0.1% Tween-20 (TBST) and 5% (*w*/*v*) non-fat dry milk. Primary antibodies were applied overnight at 4 °C: rabbit anti-Caveolin-1 (1:3000; Invitrogen, Thermo Fisher Scientific, Waltham, MA, USA; Catalog #PA1-064) and mouse anti-β-actin (1:2000; BD Biosciences, San Jose, CA, USA; Catalog #612656) as a loading control. Following three washes in TBST, membranes were incubated with horseradish peroxidase (HRP)-conjugated secondary antibodies (1:10,000; goat anti-rabbit IgG and goat anti-mouse IgG; Sigma-Aldrich, St. Louis, MO, USA) for 1 h at room temperature. Antibody details have been mentioned in Supplementary Table S1.

Immunoreactive bands were visualized using enhanced chemiluminescence (ECL) with Femto LUCENT luminol reagent (G-Biosciences, St. Louis, MO, USA) and captured on a ChemiDoc imaging system (Bio-Rad Laboratories, Hercules, CA, USA). Band intensities were quantified by densitometry using ImageJ software, version v1.54p (NIH, Bethesda, MD, USA), and Cav-1 expression was normalized to β-actin and total protein levels. Relative protein expression was calculated as a percentage of NT control neurons.

### 4.6. Intracellular Calcium Imaging

Cytosolic calcium dynamics were monitored using ratio metric fluorescence imaging with Fura-2 AM (Thermo Fisher Scientific, Waltham, MA, USA), as previously described [25]. At DIV 13, neurons were incubated with 5 µM Fura-2 AM in HEPES-buffered extracellular solution (in mM: 135 NaCl, 5 KCl, 10 Na_2_HCO_3_, 20 HEPES, 1.2 KH_2_PO_4_, 1.2 Na_2_HPO_4_, 1.8 CaCl_2_, 1.2 MgSO_4_, 10 glucose, pH 7.4) for 30 min at 37 °C. Following loading, coverslips were washed twice and mounted in a temperature-controlled perfusion chamber (37 °C) on the stage of an inverted epifluorescence microscope Leica DMi8 (Leica Microsystems, Wetzlar, Germany) equipped with a 40× apochromatic oil-immersion objective (NA 1.30).

Fura-2-loaded neurons were alternately excited at 340 nm and 380 nm using a compact LED illumination system (CoolLED pE-300, Andover, UK) with exposure times of 100–200 ms per wavelength. Fluorescence emission at 510 nm was captured every 5 s using a 12-bit Peltier-cooled EMCCD camera Andor iXon Ultra 897 (Andor Technology Ltd., Belfast, UK). The F_340_/F_380_ ratio was calculated for individual neurons (regions of interest, ROIs) using LAS X software version 3.4.2.18368 (Leica, Germany). Changes in [Ca^2+^]_i_ are expressed as the percentage change in F_340_/F_380_ ratio relative to baseline (ΔF/F_0_). Baseline fluorescence was recorded for 2 min before agonist application. Each experiment analyzed 15–25 individual neurons per coverslip, and data represent pooled results from at least three independent culture preparations.

### 4.7. Mitochondrial Membrane Potential Measurement

Ψ_m_ was assessed using the fluorescent potentiometric dye rhodamine 123 (Rh123; Sigma-Aldrich, St. Louis, MO, USA). Rh123 is a cationic lipophilic probe that accumulates in polarized mitochondria, where self-quenching occurs at high concentrations. Upon mitochondrial depolarization, Rh123 is released into the cytosol, resulting in fluorescence dequenching and increased emission intensity. At DIV 13, neurons were loaded with 5 µM Rh123 in HEPES-buffered extracellular solution (composition as in Section 2.4) for 20 min at 37 °C in a humidified incubator. After washing, coverslips were mounted in a perfusion chamber maintained at 37 °C on the inverted microscope (Leica DMi8, Wetzlar, Germany). Rh123 fluorescence was monitored using excitation at 490 nm and emission at 537 nm (bandpass filter 530–550 nm). Images were acquired every 5 s for 25 min using a 40× objective and EMCCD camera (Andor iXon Ultra 897, Belfast, UK). At the end of each experiment, maximal mitochondrial depolarization was induced by adding 1 µM carbonyl cyanide m-chlorophenyl hydrazone (CCCP; Sigma-Aldrich, St. Louis, MO, USA), a protonophore that completely dissipates Ψ_m_. Changes in Rh123 fluorescence intensity were normalized to baseline (F/F_0_) and expressed as percentage change. An increase in Rh123 fluorescence indicates mitochondrial depolarization (loss of Ψ_m_). Data were analyzed from 20–30 individual neurons per condition across at least three independent experiments.

### 4.8. Reactive Oxygen Species Detection

Intracellular ROS generation was measured using dihydroethidium (DHE, also known as hydroethidine (Het); Thermo Fisher Scientific, Waltham, MA, USA), a cell-permeable fluorogenic probe. Upon oxidation by superoxide (O_2_^−^) and other ROS, DHE is converted to ethidium, which intercalates into DNA and exhibits red fluorescence (excitation/emission: 518/605 nm).

At DIV 13, neuron-containing coverslips were mounted in a perfusion chamber at 37 °C on the inverted microscope (Leica DMi8, Germany). After establishing a stable baseline with HEPES-buffered extracellular solution for 5 min, neurons were co-treated with 5 µM DHE and glutamate receptor agonists. DHE fluorescence was monitored using excitation at 520 nm (via a BP 530/40 nm excitation filter) and emission at 635 nm (via a BA590 barrier filter). Images were acquired every 5 s for 25 min using a 40× objective and EMCCD camera.

For *Cav1*-mCherry and mCherry-transfected neurons (which exhibit red fluorescence), ROS-induced DHE fluorescence was quantified by subtracting baseline mCherry fluorescence from total red fluorescence intensity. Changes in DHE fluorescence were normalized to baseline (ΔF/F_0_) and expressed as fold-change relative to NT neurons. Fluorescence intensity was measured from individual neuronal cell bodies [52] using LAS X software (Leica Microsystems, Wetzlar, Germany), and data represent pooled results from 20–30 neurons per condition across at least three independent experiments.

### 4.9. Glutamate Receptor Agonist Stimulation

For glutamate excitotoxicity experiments, cultures were stimulated with 100 µM L-glutamate (Sigma-Aldrich, St. Louis, MO, USA) in the presence of 10 µM glycine (Sigma-Aldrich, St. Louis, MO, USA) as a co-agonist to ensure maximal NMDA receptor activation, unless otherwise specified. For selective NMDA receptor stimulation, neurons were treated with 50 µM NMDA (Sigma-Aldrich, Merck, St Louis, MO, USA) plus 5 µM glycine in Mg^2+^-free extracellular solution. AMPA receptor-mediated responses were elicited using 50 µM AMPA (Tocris Bioscience, Bristol, UK), while KA receptor activation was achieved with 50 µM KA (Sigma-Aldrich, Merck, St Louis, MO, USA). All agonist applications were performed in HEPES-buffered extracellular solution at 37 °C.

### 4.10. Statistical Analysis

All data are presented as mean ± standard error of the mean from at least three independent neuronal culture preparations. Statistical comparisons between two groups were performed using unpaired two-tailed Student’s *t*-test. Statistical analyses were conducted using OriginPro 2019b (OriginLab Corporation, Northampton, MA, USA). A *p*-value ≤ 0.05 was considered statistically significant. For all experiments, *n* represents the number of individual neurons analyzed, with each experiment repeated using neurons from at least three separate culture preparations to ensure biological replicates.

## 5. Conclusions

This study demonstrates that Caveolin-1 attenuates excitotoxic signaling in hippocampal neurons by regulating NMDA, AMPA, and kainate receptor-mediated Ca^2+^ influx at the plasma membrane. Cav-1OE significantly reduces glutamate-induced Ca^2+^ elevation, ROS generation, and mitochondrial depolarization, establishing Cav-1 as a constitutive regulator of ionotropic glutamate receptor-mediated Ca^2+^ influx during excitotoxic signaling. Future in vivo studies, together with detailed investigation of Cav-1 interactions with ionotropic glutamate receptor subunits, will be critical for translating these findings into strategies to attenuate excitotoxic neuronal injury.

## Data Availability

The data that support the findings of this study are available from the corresponding authors upon request.

## Supplementary Materials

The following supporting information can be downloaded at: https://www.mdpi.com/article/10.3390/ijms27125637/s1.

## Abbreviations

The following abbreviations are used in this manuscript:

AMPA	α-amino-3-hydroxy-5-methyl-4-isoxazolepropionic acid
AMPAR	AMPA receptor
Ara-C	Cytosine β-D-arabinofuranoside
AUC	Area under the curve
Cav-1	Caveolin-1
Cav-1KD	Caveolin-1 Knockdown
Cav-1OE	Caveolin-1 Overexpression
CCCP	Carbonyl cyanide m-chlorophenyl hydrazone
CSD	Caveolin scaffolding domain
DHE	Dihydroethidium
DIV	Days in vitro
EMEM	Eagle’s Minimum Essential Medium
ER	Endoplasmic reticulum
FBS	Fetal bovine serum
HBSS	Hanks’ balanced salt solution
HS	Horse serum
LTP	Long-term potentiation
LTD	Long-term depression
MAP2	Microtubule-associated protein 2
MOI	Multiplicity of infection
NMDA	N-methyl-D-aspartate
NMDAR	NMDA receptor
NOX	NADPH oxidase
NT	Non-transfected
ROS	Reactive oxygen species
Rh123	Rhodamine 123
ROI	Region of interest
SEM	Standard error of the mean
shRNA	Short hairpin RNA
Sc shRNA	Scrambled shRNA
Ψ_m_	Mitochondrial membrane potential
[Ca^2+^]_i_	Intracellular calcium concentration
