# Supplementary material for: Caveolin-1 Attenuates Excitotoxic Signaling by Regulating NMDA, AMPA, and Kainate Receptor-Mediated Calcium Influx in Hippocampal Neuronal Cultures
Source: Int J Mol Sci. 2026 Jun 22;27(12):5637. doi: 10.3390/ijms27125637 (PMC13300406; doi:10.3390/ijms27125637)
Supplement: Supplementary file 1 [file ijms-27-05637-s001.zip › ijms-4297095-supplementary.pdf]

Supplemental Data:

Figure S1: Overexpression of Cav-1 in hippocampal neurons.

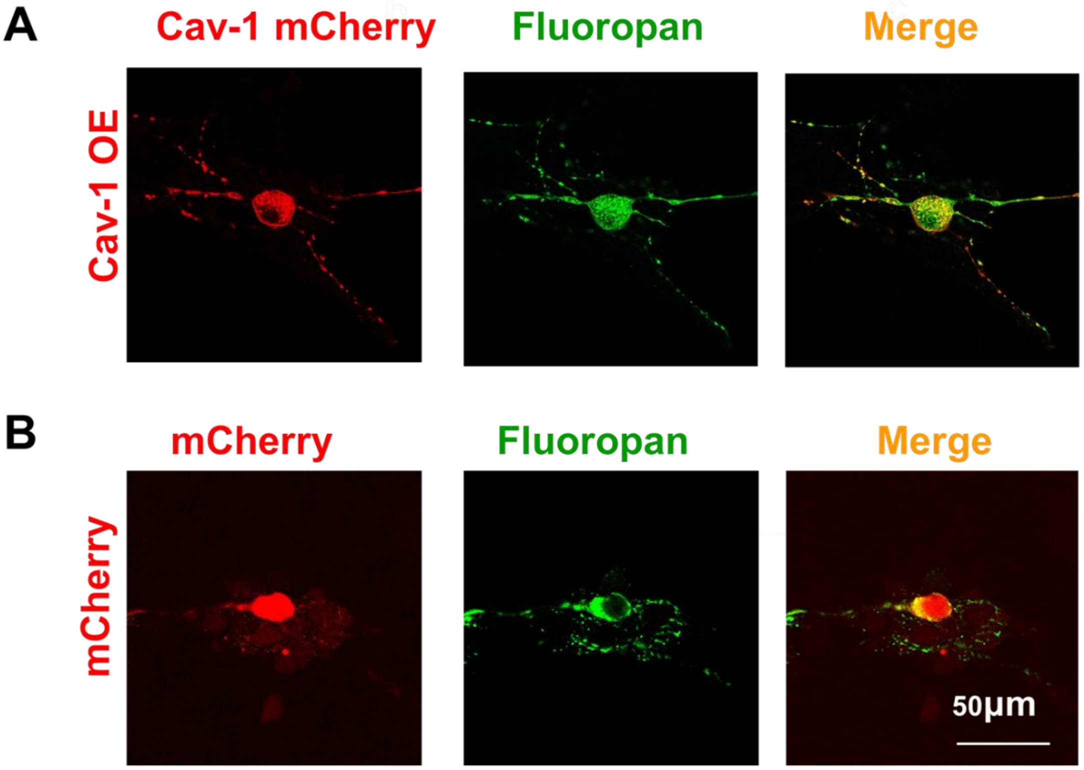

(A, B) Representative fluorescence images of hippocampal neurons transfected with either Cav-1-mCherry or empty mCherry vector (both shown in red) and labeled with the neuronal marker FluoroPan (green).

Figure S2: Only the Cav-1 overexpressed neurons inhibits [Ca2+]i response on glutamate stimulation in hippocampal neurons .

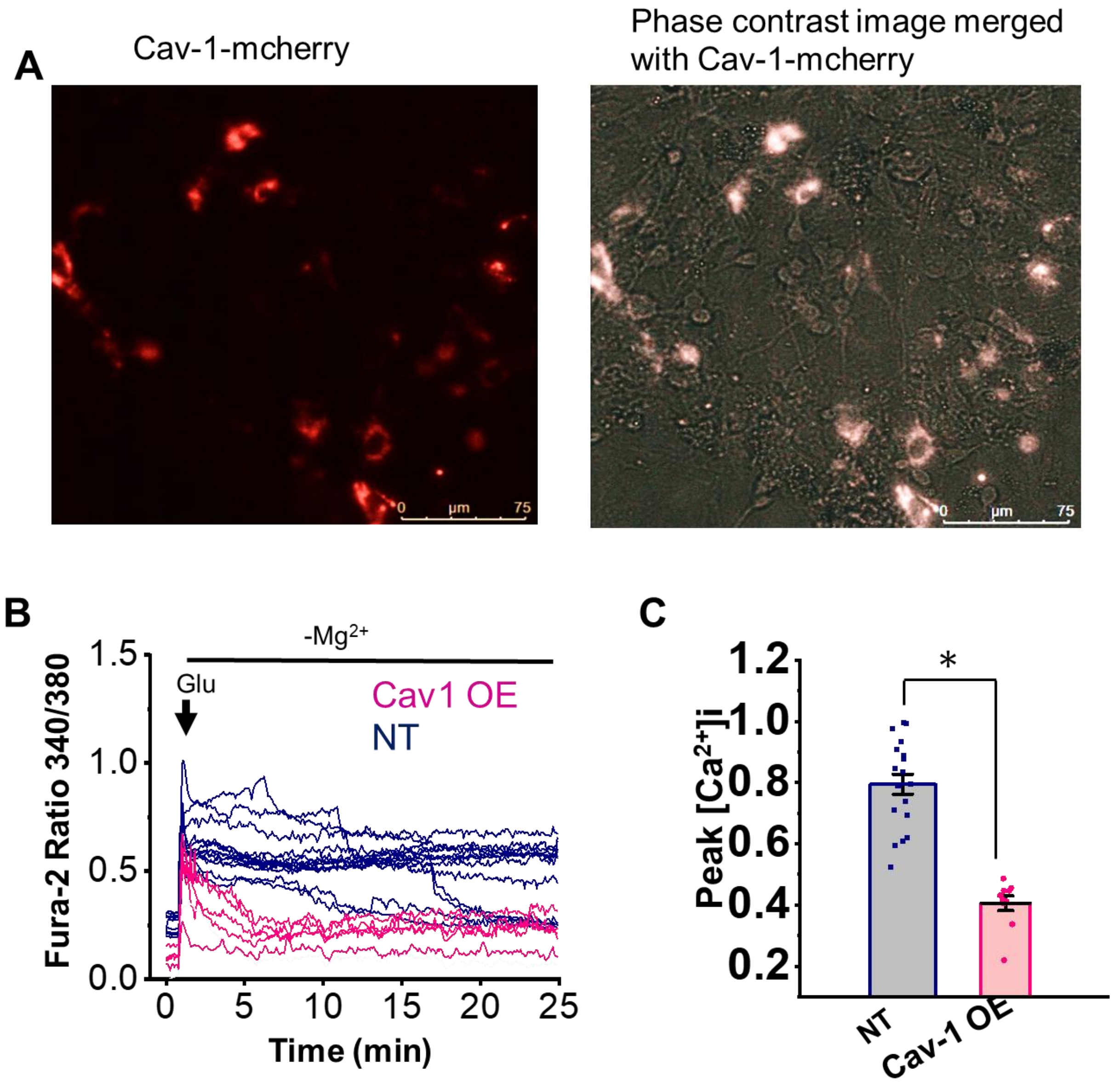

(A, B) Representative fluorescence images of hippocampal neurons transfected with Cav-1-mCherry (shown in red) and fluorescence images merged with phase contrast image. (*B*) Representative Fura-2 AM fluorescence traces showing $[Ca^{2+}]_i$ dynamics in neurons exposed to glutamate (chronic exposure, 25 min) in Mg^{2+}-free conditions for, Cav-1 overexpressing (Cav-1 OE), (*D*) Dot plots quantifying peak $[Ca^{2+}]_i$ levels. Data are presented as mean ± SEM from three independent experiments (N = 3). *$p < 0.05$ compared to NT group.

Figure S3: ShRNA mediated knockdown of Cav-1 in hippocampal neurons.

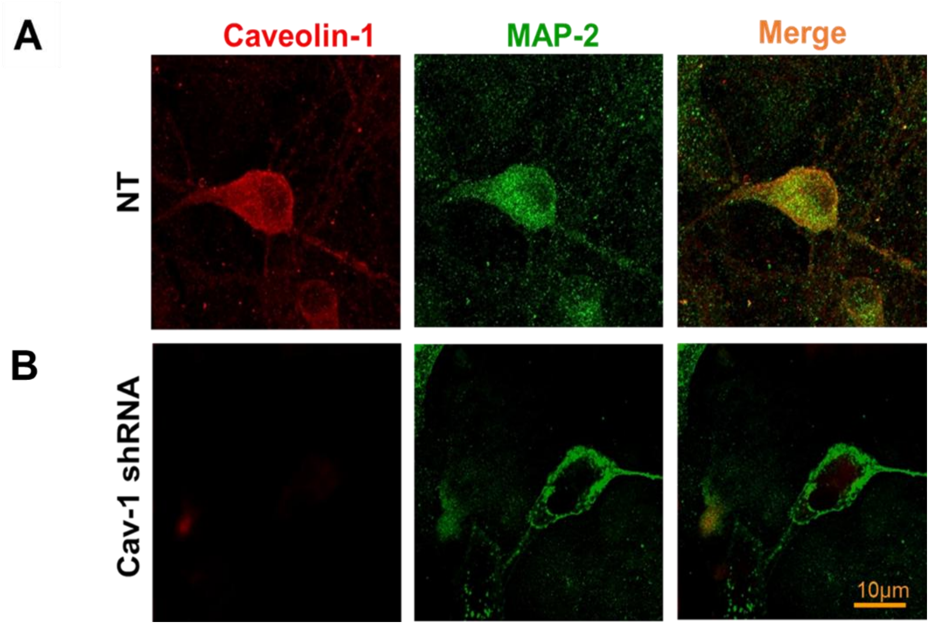

(A, B) Representative fluorescence images of hippocampal neurons infected with ShRNA Cav-1 with and NT labeled with the Cav-1, neuronal markerMAP-2 (green).

Figure S4: Western blot analysis for Cav-1 OE : Original images for WB data

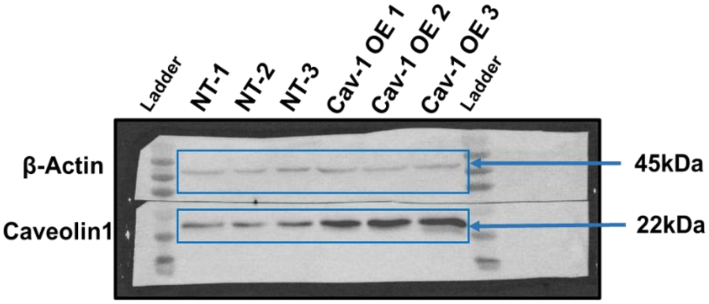

NT: Non transfected
Cav-1 OE: Caveolin 1 Overexpression

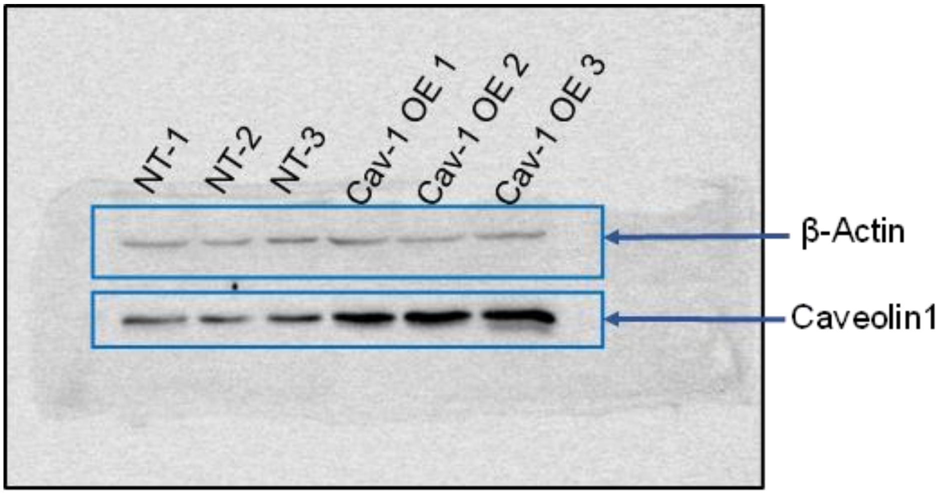

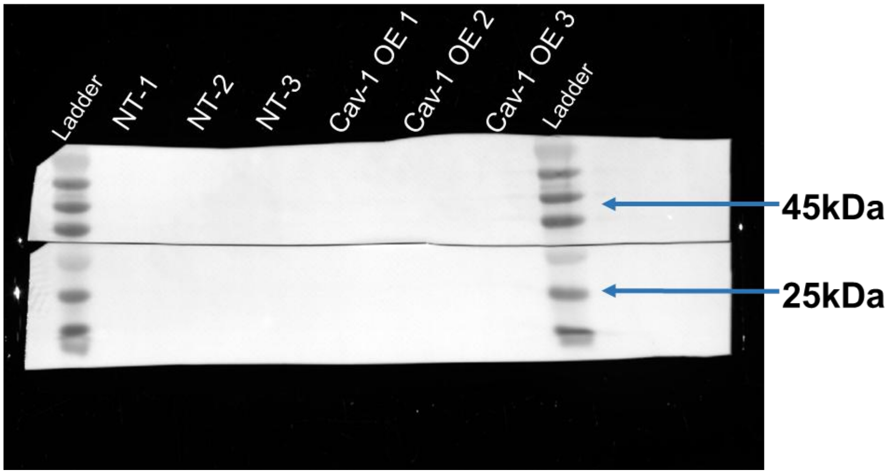

Figure S5: Western blot analysis for Cav-1 KD : Original images for WB data

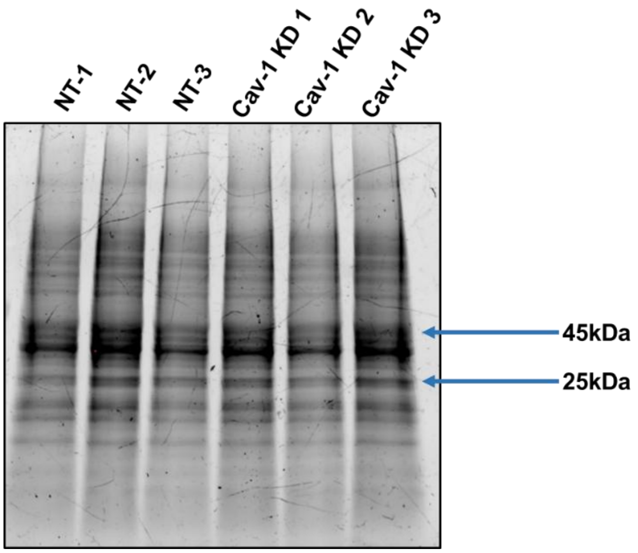

Figure: Gel staining image of total protein

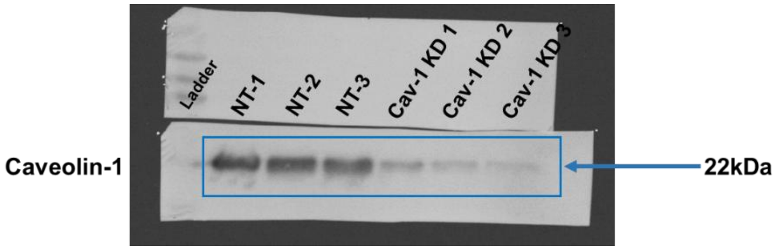

NT: Non- transfected
Cav-1 KD: Caveolin-1 Overexpression

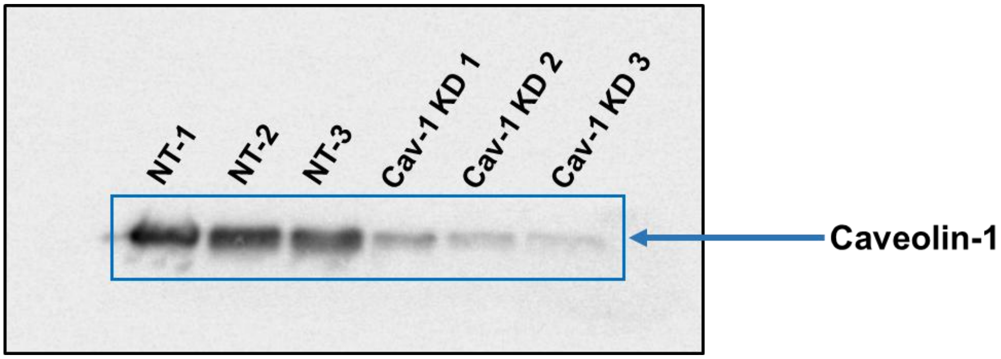

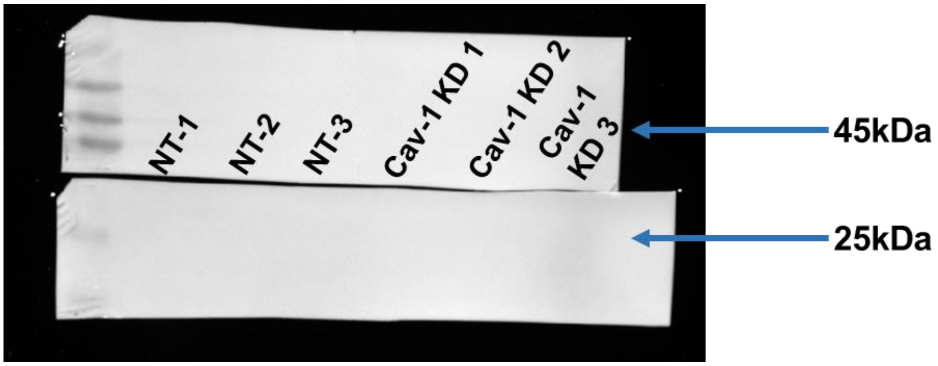

Figure S6: ROS generation during glutamate-induced excitotoxicity in neurons

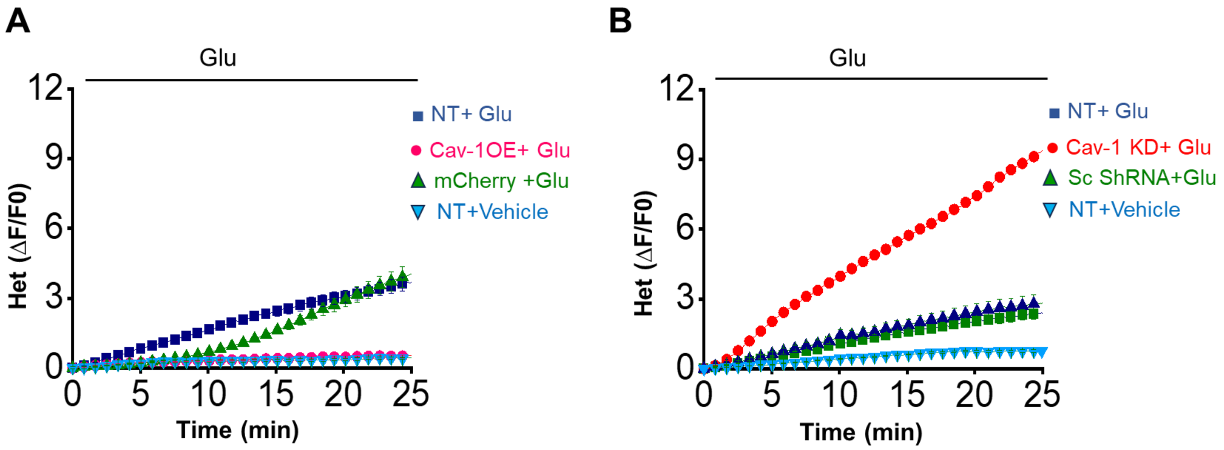

*(*A) Average ROS traces of Het fluorescence intensity indicating ROS formation in response to glutamate stimulation in NT, Cav-1 OE, mCherry-only transfected and vehicle groups. (B) Average ROS traces in NT, Cav-1 KD, Sc shRNA transduced and vehicle groups.

Figure S7: Fluorescence traces for Ψ_m depolarization in hippocampal neurons

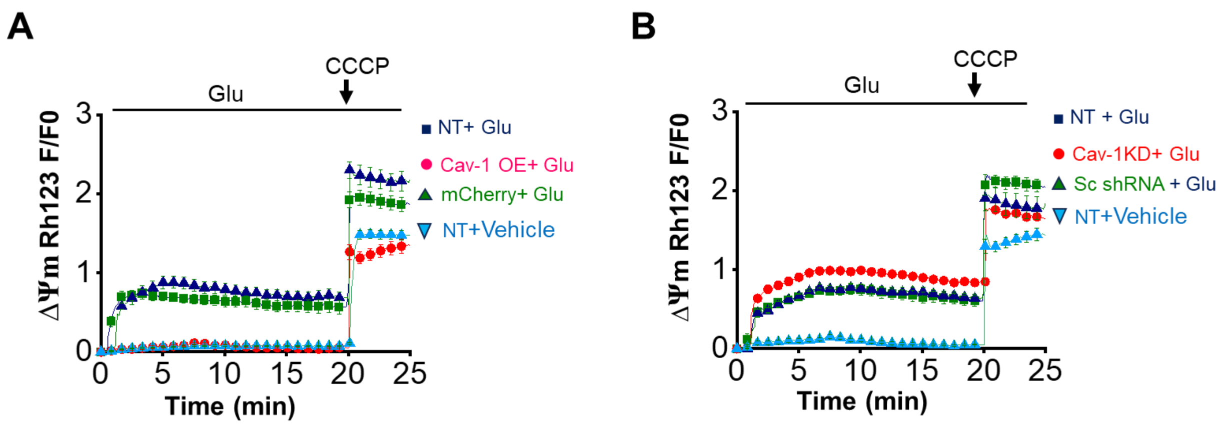

(A) Average Rh123 fluorescence traces showing changes in Ψ_m during chronic glutamate exposure (100 μM) in NT, Cav-1 OE, mCherry-only transfected and NT + vehicle only neurons. Arrowheads indicate the time of glutamate application. (B) Average Rh123 fluorescence traces in NT, Cav-1 KD, Sc shRNA transduced and NT + vehicle neurons.

Figure S8: AUC for glutamate stimulation to NT neurons

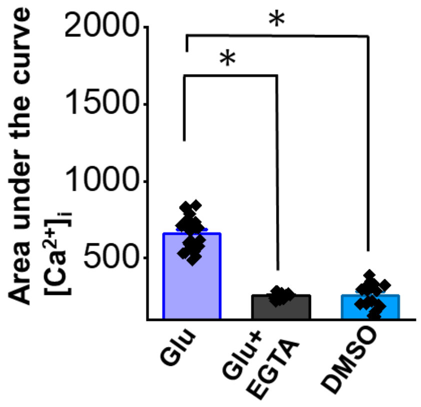

Dot plot showing AUC in NT neurons under 5 minutes glutamate stimulation (no Mg^{2+}), followed by 20 minutes perfusion with Mg^{2+}-containing buffer. Conditions include: Glu (♦, n = 31), EGTA-treated (1 mM; ●, n = 16), and DMSO vehicle (■, n = 20). Data are presented as mean ± SEM from ≥ 3 independent experiments. * $p < 0.05$; n.s. = not significant.

Table S1:

Antibody Brand	Cat. No.	Concentration
Rabbit anti-GluR2, Alomone Labs	Cat# AGC-005	IF: 1:100, western blot 1:500
Mouse anti-Caveolin-1, Santa Cruz Biotechnology	Cat# sc-53564	IF: 1:100
Mouse anti-β-Actin BD Biosciences	Cat#612656	WB: 1:2000
Mouse anti- Caveolin 1, Invitrogen, Thermo Fisher Scientific, USA	Cat#MA3-600	WB: 1:1000
Rabbit anti-Caveolin-1, Invitrogen, Thermo Fisher Scientific, USA	Cat#PA5-1064	WB: 1:1000
Rabbit anti-GluN1 (NMDAR1), Alomone Labs	Cat# AGC-001	IF: 1:100, western blot 1:500
Rabbit anti-PSD-95, Santa Cruz, USA	Cat# MA1-045	WB: 1:2000
Rabbit anti-nNOS ,Invitrogen, Thermo Fisher Scientific, USA).	Cat# N2280	WB: 1:2000
Mouse anti-MAP2, BD Biosciences	Cat# 556320	IF: 1:100
goat antimouseIgG horseradish peroxidase, Sigma, Merck, USA	Cat# A4416	1:10,000
goat anti-rabbit horseradish peroxidase, Sigma, Merck, USA	Cat# A0545	1:10,000
Alexa Fluor 488 Goat Anti-Mouse IgG, Thermo Fisher Scientific, USA	Cat# A-11001	IF: 1:500
Alexa Fluor 488 Goat Anti-Rabbit IgG, Thermo Fisher Scientific, USA	Cat# A-11008	IF: 1:500

Alexa Fluor 633 Goat Anti-Mouse IgG, Thermo Fisher Scientific, USA	Cat# A-21052	IF: 1:500
Alexa Fluor 633 Goat Anti-Rabbit IgG, Thermo Fisher Scientific, USA	Cat# A-21070	IF: 1:500
